# Laboratory biosafety: A visual analysis in the web of science database from 2000 to 2022: A review

**DOI:** 10.1097/MD.0000000000040791

**Published:** 2024-12-13

**Authors:** Sunyun Qi, Siyuan Chen, Dries De Witte, Geert Molenberghs, Qifeng Zhang, Hua Gu, Yanchao Gao

**Affiliations:** a Center for Medical Science Technology and Education Development, Hangzhou, China; b Independent Researcher, Jingdezhen, China; c Interuniversity Institute for Biostatistics and statistical Bioinformatics(I-BioStat), KU Leuven, Leuven, Belgium; d Data Science Institute(DSI), Interuniversity Institute for Biostatistics and Statistical Bioinformatics(I-BioStat), Hasselt University, Hasselt, Belgium; e Institute of Basic Medicine and Cancer, Chinese Academy of Sciences, Hangzhou, China.

**Keywords:** CiteSpace, laboratory biosafety, laboratory biosecurity, visualization, VOSviewer

## Abstract

To conduct a visual analysis of institutional publications, individual publications and publication keywords in the field of laboratory biosafety using the Web of Science database from 2000 to 2022.VOSviewer 1.6.18 was used to study the relation between paper authors, and CiteSpace 6.1.R6 was used to visualize the collaboration between the paper institutions, the paper keywords and the timeline. The main research institutions included the Centers for Disease Control and Prevention (USA), and the University of Chinese Academy of Sciences (China). The collaboration between the research institutions was limited and dispersed. Each of the main study teams is led by Feldmann Heinz, Peter B. Jahrling, Roger Hewson, and Li Na. Infection, identification, and outbreak are the keywords that appear more often and are also of higher importance in publications. The citation burst of keywords varies over time: outbreak, resistance, and polymerase chain reaction from 2004 to 2012; gene, cells, and Ebola from 2013 to 2017; and spread, safety, coronavirus, and African swine fever from 2018 to 2022. The centralization of research teams and individuals in laboratory biosafety is not conducive to the growth of disciplinary diversity. These publication keywords are mainly align with significant social events, scientific and technological development trends, and national strategic needs. This paper advocates for a more balanced allocation of resources and collaboration opportunities to foster diversity and address emerging challenges in the field of laboratory biosafety.

## 1. Introduction

Biosafety refers to the statutes and capacity to effectively respond to hazards or prospective risks to the national society, economy, public health, and ecological environment posed by bio-related factors.^[1]^ In the early 20th century, developed countries in Europe and the United States (US) recognized the importance of biosecurity to national security by identifying biosecurity, nuclear security, and cyber security as the 3 major national security concerns.^[2]^ As globalization continues to advance, biosafety may have a greater impact than nuclear and chemical threats due to its persistent nature, diverse sources, and borderlessness,^[3]^ which poses a significant threat to human life and health as well as social and economic development on a global scale. It has become one of the most prominent threat to national and strategic security,^[4]^ and as a result, the prevention of biological hazards has become the primary concern of the Western world. Since the 1970s, the US has enacted laws and regulations such as *Public Health Security* and *Bioterrorism Preparedness and Response Act*,^[5]^ *the National Biodefense Strategy and Implementation Plan for Countering Biological Threats, Enhancing Pandemic Preparedness, and Achieving Global Health Security*. The United Kingdom released its *Biological Security Strategy* in 2018. Russia developed the *National Security Strategy* in 2021^[6]^ and China introduced the *Biosecurity Law of the People’s Republic of China* in 2021.^[7]^

Laboratory biosafety, an important component of biosafety, is a vital resource for the prevention and control of new and emerging infectious diseases, a key platform for cutting-edge technological research, and an essential strategic national reserve.^[8]^ However, events such as the US anthrax mailings in 2001,^[9]^ SARS-CoV-1 in 2003,^[10]^ SARS-CoV-2 induced COVID-19 pandemic, the scandal of lost deadly strains such as anthrax in Fort Detrick revealed in 2021, and coronavirus traceability have made biosafety laboratories extremely controversial.^[11]^ Due to its importance and the significant dangers posed by unanticipated events, laboratory biosafety has become a focal point and a popular topic in academia. However, there is a paucity of comprehensive studies that systematically analyze the global landscape of laboratory biosafety research. This study aims to identify key research hotspots, the evolution of research focus, and the potential for interdisciplinary collaboration by utilizing the Web of Science (WoS) database from 2000 to 2022. By understanding key topics, active collaboration areas, and emerging trends, this study on laboratory biosafety seeks to provide researchers and policymakers with a clear picture of the current research landscape. Moreover, it serves as a guide for directing efforts and resources to the most impactful areas, ensuring that laboratory biosafety remains a priority and continues to evolve in response to new challenges.

## 2. Materials and methods

### 2.1. Data source

From 2000 to 2022, significant advancements in biotechnologies such as gene editing, the emergence of new biological threats and potential bioterrorism risks, the frequent occurrence of global public health events, and the improvement of laboratory infrastructure have collectively heightened global awareness and concern for laboratory biosafety. The WoS database was selected for our study due to its extensive coverage across multiple fields and a vast collection of peer-reviewed articles. Moreover, it enables us to effectively assess the impact of various research papers and conduct a bibliometric study. The literature data were obtained on February 13, 2023, from the Science Citation Index Expanded (SCI-EXPANDED) database of the WoS Core Collection. To reduce potential bias induced by database upgrades, all searches were performed on the same day. English is the dominant language in scientific communication, and a significant portion of research publications are written in English. Therefore, our refining method included the following: (1) the advanced search formula was configured as TS = (“laborator* biosafety” or “laborator* biosecurity”). (2) Limiting the document classification to “ARTICLE” or “REVIEW.” (3) Articles were written in English. (4) Articles published between 2000 and 2022 were retrieved. Finally, 1209 references were collected. We saved the document information as comprehensive records, including the abstract, keywords, author, institution, and country data and cited references in plain text format. Therefore, these 1209 references constituted the final literature database.

### 2.2. Methods

CiteSpace and VOSviewer, 2 well-known scientometric analysis tools, were used to visualize trends in laboratory biosafety literature. CiteSpace enables the identification of key papers, authors, and research fronts through citation network visualization.^[12]^ Meanwhile, VOSviewer displays bibliometric networks, making it easier to investigate theme structures and collaboration networks.^[13]^

CiteSpace 6.1.R6 was used to visualize the collaboration between the institutions, the keywords, and the timeline of the articles, with the time span from January 1, 2000, to December 31, 2022, using a time slice of 1 year. TOPN was set to be 50 in the CiteSpace process, indicating that the top 50 most cited or appearing items from each slice were chosen, with time, institutions, and keywords as the nodes, respectively.^[14]^ The log-likelihood ratio clustering algorithm was chosen for the clustering analysis.^[15]^

VOSviewer 1.6.18 was used to analyze the relation between the authors of the papers. In the VOSviewer, the minimum number of documents for an author was set to be 3, as commonly chosen, and 130 authors met this criterion. For each of the 130 authors, the total strength of the co-authorship links with other authors was calculated. The authors who had the highest total link strength were selected. The annual number of published articles was processed using R 4.2.2.

## 3. Results

### 3.1. Annual quantitative distribution of publications

**Figure 1.**
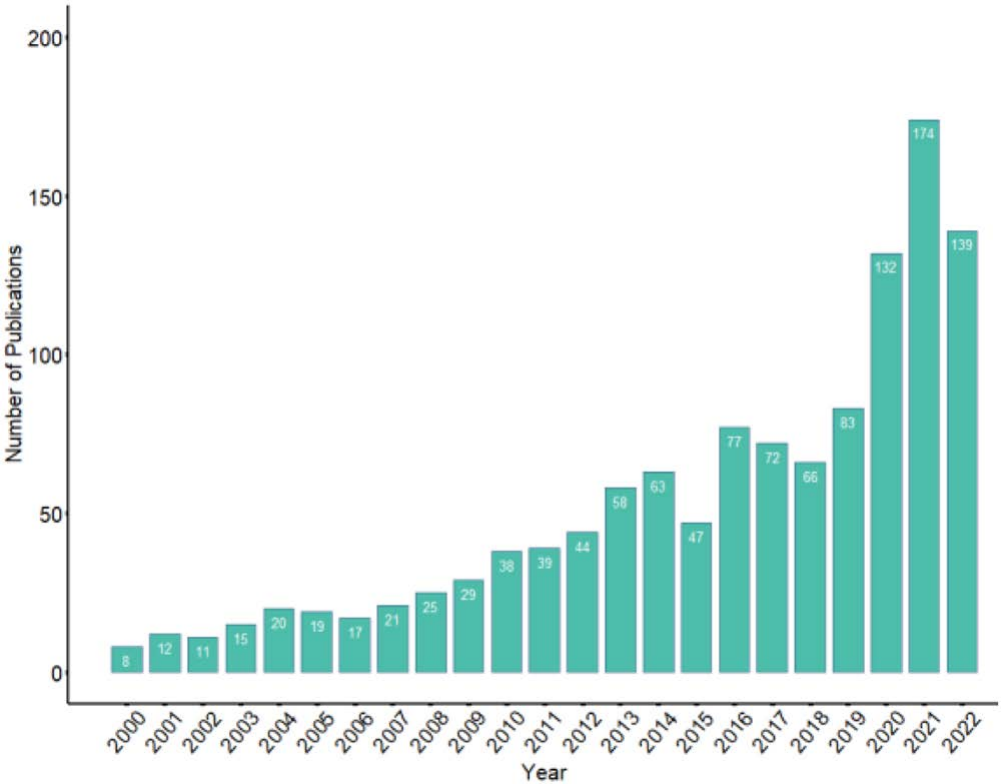
Annual distribution of the articles related to laboratory biosafety published from 2000 to 2022. The rising trend highlights growing attention to biosafety research.

Between January 2000 and December 2022, a total of 1209 articles on laboratory biosafety were published. In Figure 1, the number of publications in each year is given. We can conclude that the field of laboratory biosafety has gained progressively more attention over the course of time, as the number of articles published in these years exhibits an increasing trend. Major events in the field of laboratory biosafety, such as US anthrax in 2001, SARS-CoV-1 in 2003, avian influenza in 2005, African swine fever in 2008, influenza A in 2009, MERS in 2013, and COVID-19 in 2019, increased the academic community’s focus on laboratory biosafety. Specifically, 174 articles were published in 2021, the year with the highest number of publications. Ultimately, this sustained growth signifies that the field is receiving the attention it merits, which is essential for advancing scientific research and ensuring the safety of all involved.

### 3.2. Institutions analysis

Table 1 presents the ten institutions with the most publications on laboratory biosafety from January 2000 to December 2022. The top 10 institutions in terms of the number of laboratory biosafety-related articles published are the Centers for Disease Control and Prevention (CDC, USA, 37 articles), University of Chinese Academy of Sciences (China, 28 articles), National Institute of Allergy and Infectious Diseases (USA, 26 articles), Friedrich Loeffler Institute (German, 13 articles), University of California, Davis (USA, 12 articles), University of Texas Medical Branch (USA, 12 articles), Aix Marseille University (France, 11 articles), Bernhard Nocht Institute for Tropical Medicine (German, 11 articles), Public Health England (UK, 10 articles), and Emory University (USA, 9 articles). The most influential authorities in laboratory biosafety are mainly located in the US and Europe countries. The US and several European countries host a significant concentration of influential laboratory biosafety research, largely driven by robust research and development investments, a well-established scientific infrastructure, and advanced facilities that support innovative work in this field. Meanwhile, China has made notable progress in recent years through increased investments in laboratory biosafety, improvements to its research infrastructure, and dedicated efforts to cultivate expertise in the field.

**Table 1:** Top 10 institutions of publications about laboratory biosafety.

Rank	Institution	Country	# of publication
1	Centers for Disease Control and Prevention	USA	37
2	University of Chinese Academy of Sciences	China	28
3	National Institute of Allergy and Infectious Diseases	USA	26
4	Friedrich Loeffler Institute	German	13
5	University of California, Davis	USA	12
5	University of Texas Medical Branch	USA	12
7	Aix Marseille University	France	11
7	Bernhard Nocht Institute for Tropical Medicine	German	11
9	Public Health England	UK	10
10	Emory University	USA	9

**Figure 2.**
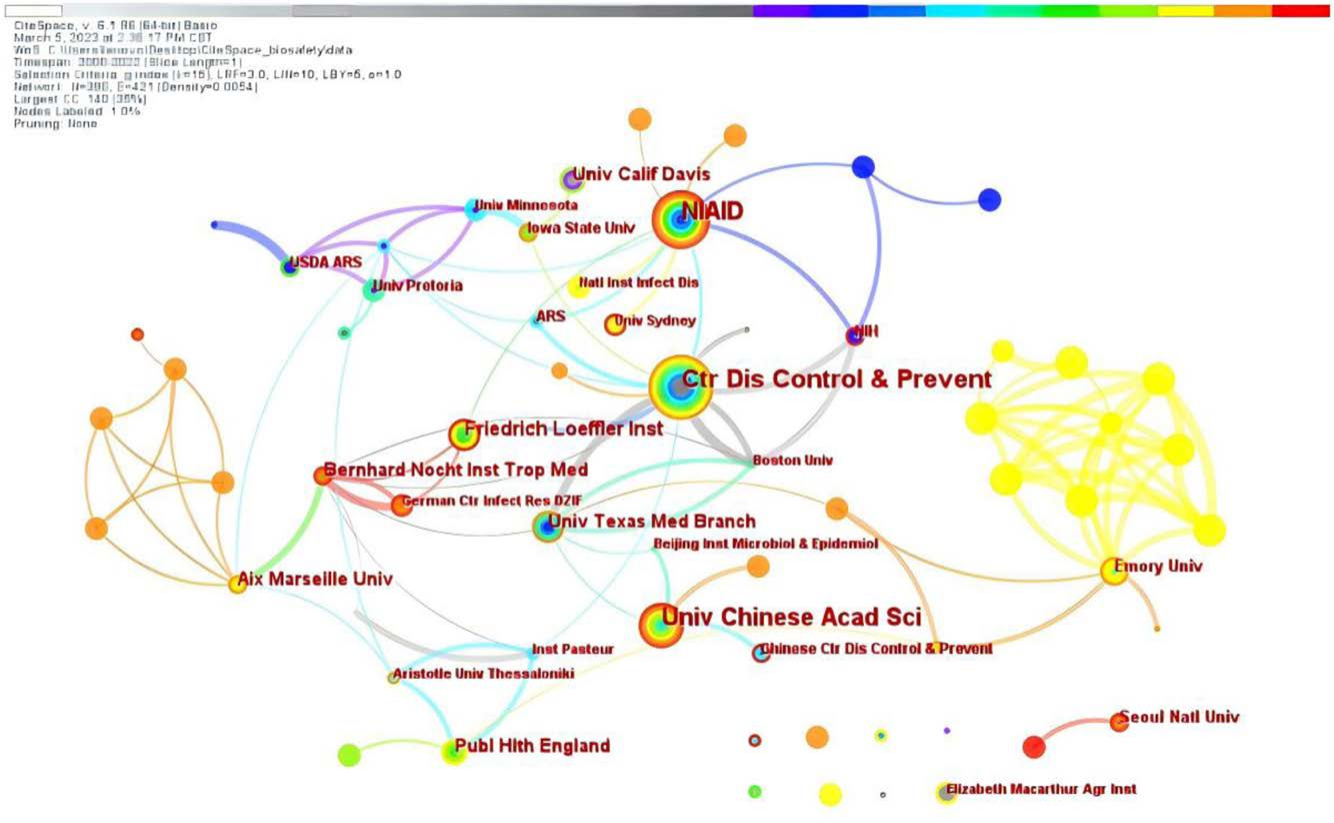
Collaborative network of institutions of publications about laboratory biosafety from 2000 to 2022. A circle node represents the institution of papers; a line between nodes represents partnership. It highlights key institutions such as the Centers for Disease Control and Prevention (CDC) in the United States and the University of Chinese Academy of Sciences in China, which are leading a global research initiative.

Figure 2 depicts the institutional collaboration with 396 nodes, 421 lines, and a network density of 0.0054. Each node represents a single institution, and the size of the node’s circle indicates the number of articles published by that institution. The thickness of the line between nodes reflects the degree of collaboration between institutions. CDC, which is ranked first in terms of the number of publications, collaborates with a larger number of institutions, but the frequency of collaboration with individual institution is comparatively low (Fig. 2). The University of Chinese Academy of Sciences collaborates closely not only with the Beijing Institute of Microbiology and Epidemiology, Tsinghua University, and the Chinese CDC, but also with the University of Texas Medical Branch.

### 3.3. Authors analysis

**Figure 3.**
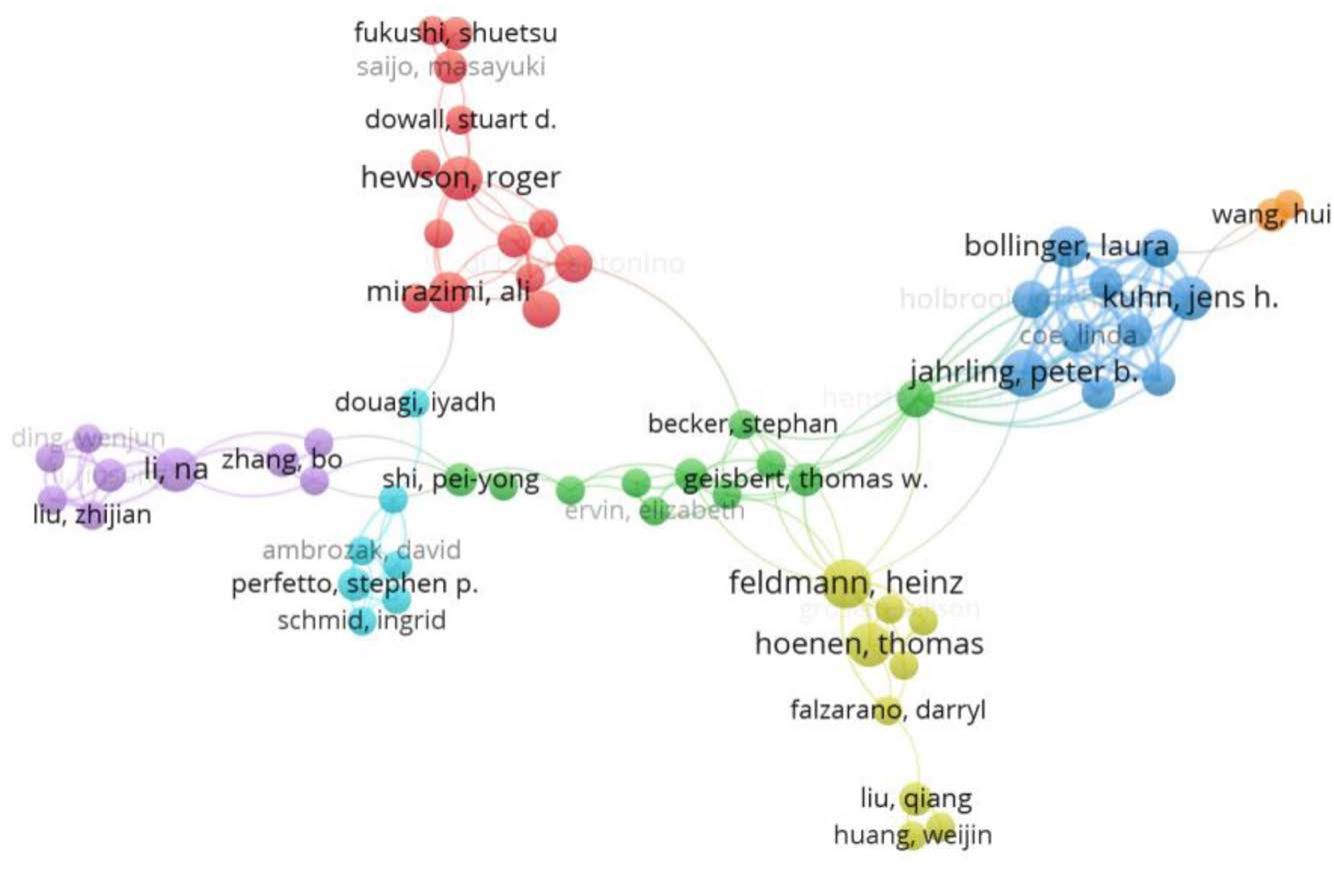
Collaborative network of authors of publications about laboratory biosafety from 2000 to 2022. A circle node represents the authors of papers; a line between nodes represents partnership. It reveals the collaborative efforts of researchers that extend across continents and institutions.

From January 2000 to December 2022, WoS lists a total of 7758 authors of publications related to laboratory biosafety. Benelli Giovanni has the highest number of publications, with a total of 9. Figure 3 shows the authors’ collaborative network of authors with 62 nodes and 176 lines. Each node in the map represents an individual author, and the size of the node’s circle indicates the number of articles published by that author. The line connecting the nodes symbolizes collaboration. It can be concluded that there are 7 major research teams. Considering the scale of collaboration and the number of publications, there are 4 teams (1) with Feldmann Heinz as the core, Hoenen Thomas, Falzarano Darry and others; (2) with Jahrling Peter B. as the core, Bollinger Laura, Kuhn Hens H. and others; (3) with Hewson Roger as the core, Mirazimi Ali, Fukushi Shuetsu and others; (4) with Li Na as the core, Zhang Bo, Liu Zhijian and others. In terms of publication time, the team with Li Na as the core and the team with Shi Pei-Yong, Ervin Elizabeth as the core have an increase in publications between 2019 and 2022, indicating that these 2 teams have conducted cutting-edge research during this time period. The concentration of research within a few teams suggests risks such as research homogenization, uneven resource distribution, inadequate information exchange, and increasing barriers to entry in laboratory biosafety. These issues are detrimental to the sustainable development and diversification of the discipline.

### 3.4. Research hotspots and frontier trend analysis

#### 3.4.1. High-frequency keywords

**Figure 4.**
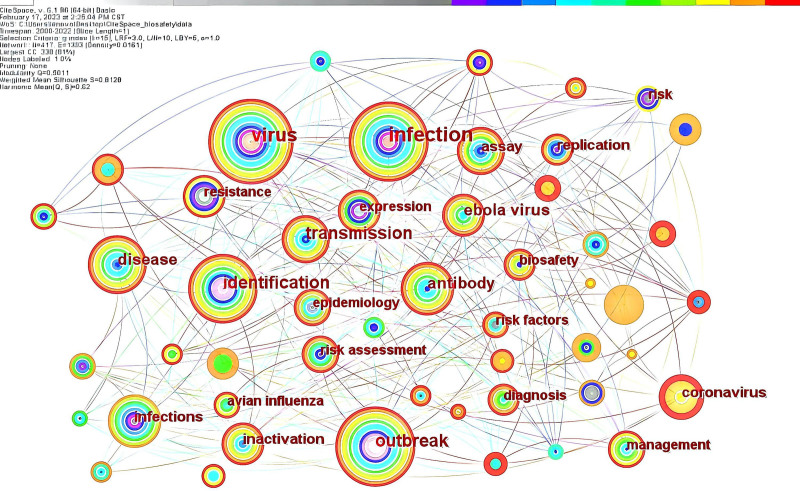
Network of paper keywords in the field of laboratory biosafety from 2000 to 2022. Circle nodes represent the keywords of papers; the color of circles and lines between nodes represents time. Keywords such as “virus,” “infection,” and “transmission” signify ongoing areas of interest, while the color gradient illustrates the temporal evolution of research topics.

Figure 4 shows the network map of paper keywords using CiteSpace. It has 417 nodes, 1393 lines, and a network density of 0.0161. Each node represents a keyword, and the size of the node’s circle indicates the frequency of the keyword. The color of the node circles and connecting lines represents time, with red representing recent years and gray referring to points more distant in the past. The frequency of the keywords can reflect the research hotspots, and in our laboratory biosafety research, the top 5 keywords are infection (95 times), virus (74 times), transmission (50 times), identification (48 times), outbreak (48 times)(Table 2). The centrality value of keyword nodes reflects their influence and significance in the network. The larger the centrality is, the more influential and important the keyword is. The 5 most important keywords are virus (0.20), infection (0.19), outbreak (0.16), identification (0.13), and antibody (0.11) (Table 3). The prominence of keywords such as infection, viruses, transmission, identification, and outbreaks underscores their significance as central themes in laboratory biosafety research. This trend indicates that research hotspots are aligned closely with urgent societal issues. However, it also reveals potential research gaps, particularly in areas such as risk assessment and safety governance, which may not be receiving sufficient attention.

**Table 2:** Top 5 keywords in terms of frequency.

Rank	Keyword	Frequency
1	Infection	95
2	Virus	74
3	Transmission	50
4	Identification	48
4	Outbreak	48

**Table 3:** Top 5 keywords in terms of centrality.

Rank	Keyword	Frequency
1	Virus	0.20
2	Infection	0.19
3	Outbreak	0.16
4	Identification	0.13
5	Antibody	0.11

#### 3.4.2. Keyword cluster analysis

**Figure 5.**
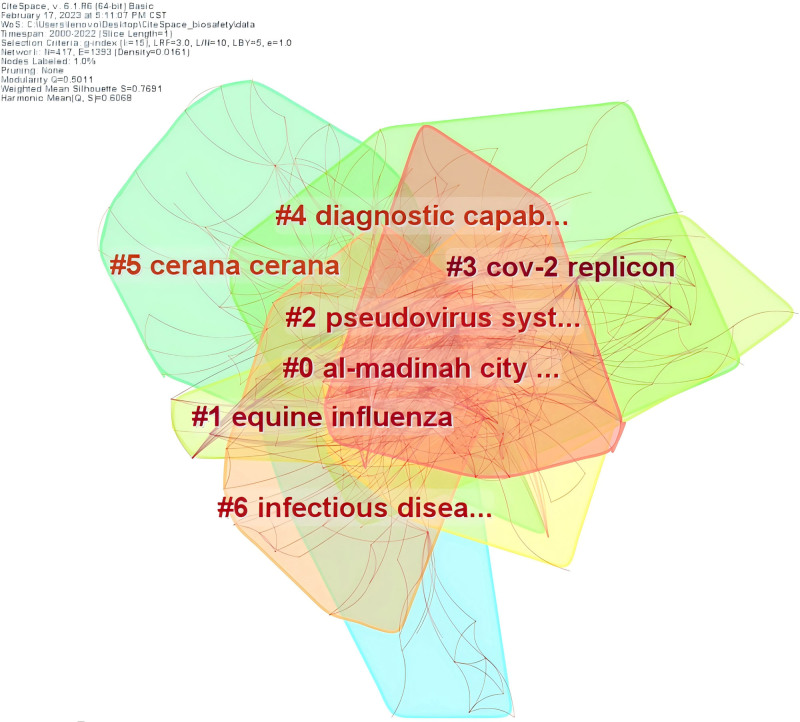
Top 7 keyword clusters. It reveals distinct research foci, with “al-madinah city saudi arabia” and “equine influenza” emerging as central themes within their respective clusters.

**Figure 6.**
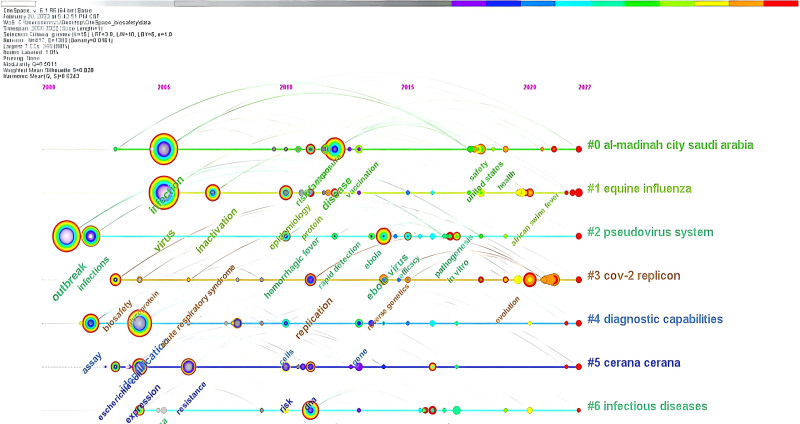
Timeline view of the top 7 keyword clusters. Each horizontal line represents a cluster. The timeline is shown at the top of the figure, and the year corresponding to the node is its publication time. “Outbreaks” and “infections” predominated in the early 2000s, shifting to “disease” in 2012, as shown in the evolving keyword clusters.

Using CiteSpace, related keywords were grouped into a single cluster. Figure 5 shows the top 7 keyword clusters and Figure 6 illustrates the chronology of these 7 clusters with 417 nodes, 1393 lines and a network density of 0.0161. The labels generated by the clusters are the centers in the field of biosafety in the laboratory. The value of the silhouette measures the cluster’s consistency and homogeneity. The closer the silhouette is to 1, the greater the cluster’s consistency. As shown in Table 4, all the values of silhouette for the top 7 keyword clusters fall within the range of 0.761 to 1.000, which means that the clusters are well consistent.

**Table 4:** Keyword clusters characteristics.

Cluster	Label	Main keywords	Silhouette
#0	al-madinah city saudi arabia	al-madinah city saudi arabia; campylobacter spp; biosecurity factor; ontario canada; demographic husbandry	0.761
#1	equine influenza	equine influenza; african swine fever; equine influenza outbreak; african swine fever virus; saliva sample	0.789
#2	pseudovirus system	pseudovirus system; ebola virus disease; marburg virus; ebola virus; replication-competent virus-like particle system	0.795
#3	cov-2 replicon	cov-2 replicon; drug evaluation; recent advance; bunyavirus reverse genetics research; future perspective	0.824
#4	diagnostic capabilities	diagnostic capabilities; plant disease; biotechnological research; safety management; dangerous pathogen	0.804
#5	cerana cerana	cerana cerana; chinese honeybee; bt-transgenic maize pollen; predator orius insidiosus; thrips tabaci	0.813
#6	infectious diseases	infectious diseases; publication proliferation; pandemic risk; influenza virus research; eu export regulation	0.831
#7	advanced hiv	advanced hiv; tuberculosis co-occurrence; broad clinical spectrum; hiv-infected patient; years experience	0.750
#8	livestock export	livestock export; mycobacterial lipoarabinomannan; rapid diagnosis; recombinant junin virus n protein; argentine hemorrhagic fever	0.802
#9	malaria vector	malaria vector; green-synthesised nanoparticle; melia azedarach seed; eco-friendly route; cyclopoid crustacean cyclops vernalis	0.932
#10	lactobacillus gasseri	lactobacillus gasseri; oral probiotic activities; potential oral probiotic properties; lactobacillus fermentum ok; biosafety evaluation	0.934
#11	two-year prospective study	two-year prospective study; ontario canada part; backyard chicken flock; strain origin; retrospective analysis	0.808
#12	Avoiding biohazard	avoiding biohazard; research laboratories; aids era; biosafety consideration; laboratory worker	0.968
#13	guideline	guideline; ivf; infection; review; biosafety level	1
#14	wild rodent	wild rodent; quarantine; outdoor facility; hantavirus; biosafety level	1
#15	Safety precaution	safety precaution; bsl-4 laboratory; operating procedure; aerobiology; suit laboratory suite entry	1
#16	Population-scale laboratory studies	population-scale laboratory studies; transgenic plant; nontarget insect; effect; biosafety level	1
#17	assessment	assessment; candidate agent; onopordum spp; thistle; australia	1
#18	single-species	single-species; microbial biofilm; industrial application; biosafety level; risk factor	1
#19	science	science; biosafety; case; transgenic crop plant; politics	1

Given Figure 6, the most popular keywords were outbreaks and infections (#2 pseudovirus system) in 2000, infections (#0 al-madinah city saudi arabia, #1 equine influenza) and glycoprotein (#4 diagnostic capabilities) in 2005, and disease (#0 al-madinah city saudi arabia) in 2012.

In Table 4, the keyword cluster for the city of Al Madinah, Saudi Arabia keyword cluster is dominated by terms related to infectious diseases and biosafety concerns due to religion. The keywords for the equine influenza cluster focus on the outbreak of animal virus. The majority of the keywords, in the pseudovirus system cluster are related to transcription of Ebola and Marburg virus variants. The keywords for the cov-2 replicon cluster focus on the replicon, drug evaluation related to the replicon and future prospects. Biotechnological research to identify threat factors, such as plant diseases and hazardous pathogens, is the focus of the diagnostic capabilities keyword cluster. The keywords for the cluster cerana focus primarily on its mediating function and diversity. The cluster of keywords for infectious diseases focuses on the study of the risk of epidemic virus transmission. The studies indicate that research in laboratory biosafety is centered on a few key areas, with these focal points evolving over time. Factors such as geography, culture, and religion, along with public health events, significantly influence shifts in research priorities. Additionally, advancements in technology and the convergence of interdisciplinary fields propel the ongoing expansion of these research hotspots. This dynamic interplay suggests that while certain core themes remain central, the landscape of biosafety research is also responsive to broader societal and scientific developments.

#### 3.4.3. Keyword citation burst

**Figure 7.**
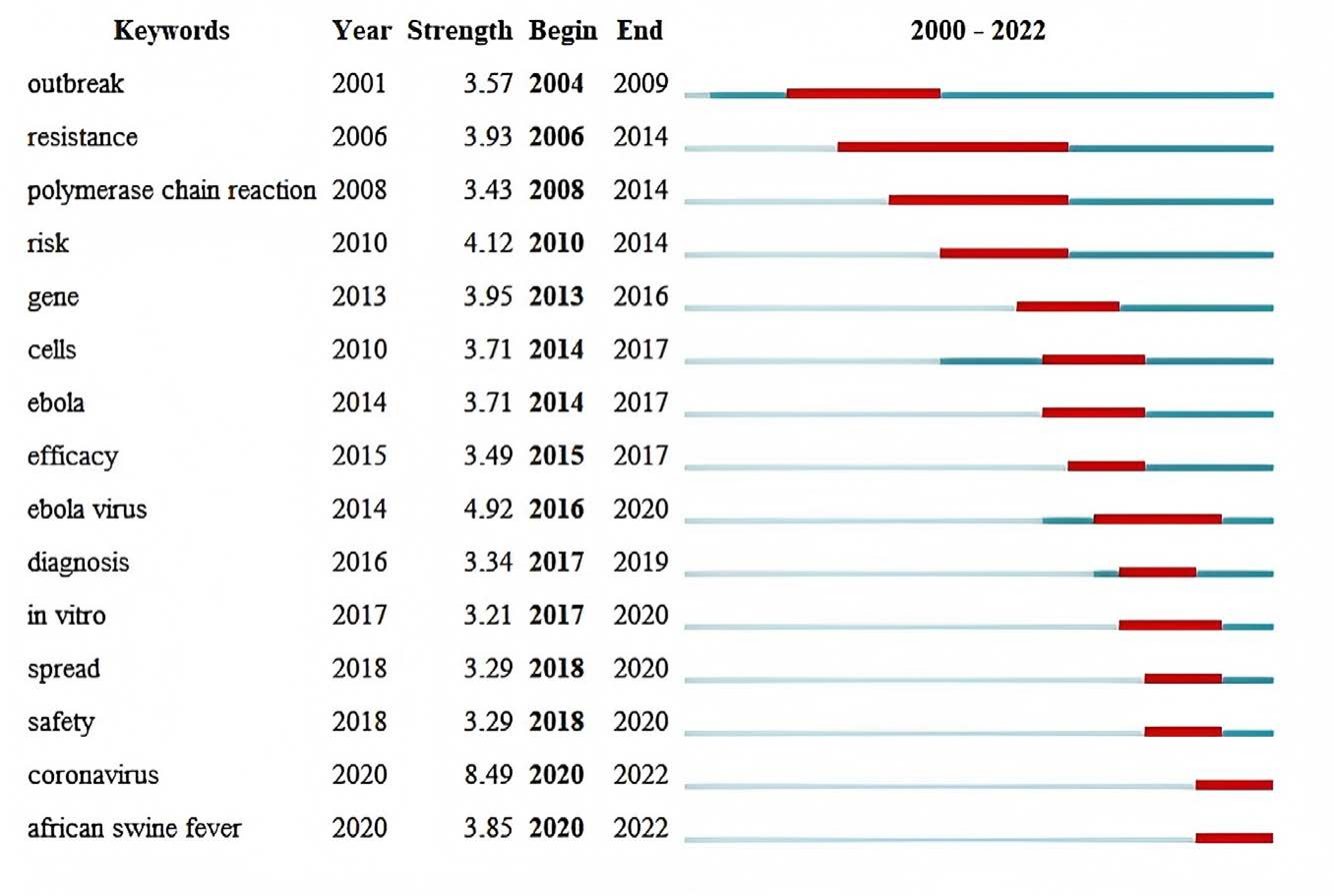
Top 15 keywords with the strongest citation bursts. It indicates significant shifts in laboratory biosafety research, with “coronavirus” experiencing the most substantial increase in attention starting from 2020.

The citation burst of keywords can detect the frontier and trend of a research field, and the burst of high-frequency keywords at various times represents the research directions that emerged and received a great deal of attention during that period, thus further reflecting the evolution process. The stronger the citation burst, the more cutting-edge the research. The top 15 keywords with the strongest citation burst were derived by CiteSpace, as shown in Figure 7. The length of the red bar indicates the duration of the keyword explosion, which represents the period’s research frontier. From 2004 to 2013 (not included), laboratory biosafety papers in WoS were mainly related to outbreak, resistance, polymerase chain reaction and risk; from 2013 to 2018 (not included), the frontiers centered on gene, cells, Ebola, efficacy, Ebola virus, diagnosis and in vitro; from 2018 to 2022, the frontiers were mainly concentrated on spread, safety, coronavirus, and African swine fever. The top 6 keywords with the strongest citation burst are coronavirus (strength = 8.49), Ebola virus (strength = 4.92), risk (strength = 4.12), gene (strength = 3.95), resistance (strength = 3.93), and African swine fever (strength = 3.85). The research underscores that assessing human health risk factors remains a top priority in laboratory biosafety. Innovations in technology and methodology have opened new research paths, while shifts in focus, interdisciplinary collaboration, and the emergence of novel research frontiers have advanced the field to a more developed stage. This highlights the ongoing need for vigilance and adaptation in laboratory biosafety to address emerging threats and challenges.

## 4. Discussion

As the landscape of laboratory biosafety research continues to shift, understanding trends is crucial for identifying gaps and opportunities for further inquiry. Our visual analysis of laboratory biosafety research from 2000 to 2022, utilizing data from the WoS database, reveals key insights into the evolution and current state. The increasing number of publications during this timeframe highlights the growing recognition of the importance of laboratory biosafety in global health security and the prevention of potential biohazards.^[16]^

The concentration of influential research in a few leading institutions, particularly in the US and Europe, highlights the geographic distribution of expertise and resources in this field. This centralization indicates that while these regions are driving much of the research agenda, there is potential for collaboration and knowledge exchange with institutions in other parts of the world.^[17]^ The institutional partnerships also reveal that CDC and National Institute of Allergy and Infectious Diseases collaborate with a larger number of institutions, underscoring their leadership and authority in the field of laboratory biosafety research in the US. Taking China as an example, the University of the Chinese Academy of Sciences serves as the center of collaboration among Chinese academic institutions. However, most of its collaborating institutions are domestic. This finding aligns with the results drawn from Zhiming Y,^[18]^ which indicate that Chinese institutions and teams lack external collaboration. Nonetheless, the centralization of research efforts raises concerns about research homogenization and the unequal distribution of resources. To cultivate a more diverse and inclusive research environment, it is essential to promote international collaboration, enhance biosafety capacities in regions that are currently underrepresented in the literature, and encourage interdisciplinary approaches to tackle the complex challenges in laboratory biosafety.^[19]^

The identification of key research teams and their areas of focus provides insights into the current research landscape. The prevalence of specific keywords such as “infection,” “virus,” “transmission,” “identification,” and “outbreak” reflects the primary concerns and priorities within laboratory biosafety. These keywords highlight the ongoing challenges in managing infectious diseases and the need for robust protocols to prevent their spread. The citation burst analysis reveals the dynamic nature of research interests, with shifts in focus over time corresponding to global health events and emerging threats.^[20]^ For instance, the notable increase in publications related to “coronavirus” post-2019 clearly responds to the COVID-19 pandemic. This trend underscores the field’s capability to adapt and respond to urgent public health crises.

Among the clustered keywords, the city of Al Madinah, Saudi Arabia, as a well-known religious site, may generate increased academic interest and concern about potential public health incidents.^[21]^ The analysis of clustered keywords, which supports the creation and development of antibodies and medications for infectious diseases, as well as cutting-edge biotechnology research, aligns with the investigation by Brouqui P.^[22]^ Thus, societal hot spots, technological advances, and national strategic requirements must be closely monitored in laboratory biosafety research.

Although we performed a comprehensive exploratory analysis and provided an accurate representation of laboratory biosafety publications, this paper is limited by limited data, which may result in a biased analysis. Specifically, only literature from the WoS was included, and only English-language articles and reviews were considered. This may overlook significant contributions from diverse regions and languages. Future research should adopt a more inclusive approach by incorporating a wider range of sources and languages to provide a more comprehensive perspective on laboratory biosafety.

## 5. Conclusion

Our research indicates that the field of laboratory biosafety is progressively maturing, with continued focus on investigating risk factors that pose threats to human health. The evolution of new technologies and the increasing integration of interdisciplinary approaches will be pivotal in the future. These advancements can foster a robust trajectory for laboratory biosafety research by establishing interdisciplinary collaboration networks, creating multi-channel information platforms, and strengthening international cooperation to address global biosafety challenges. Additionally, focusing on technological innovations and investing in infrastructure to support cutting-edge research and safety measures is crucial.

Based on these findings, we recommend that policymakers enhance global cooperation, build international research networks, and implement joint projects and collaborative research grants. Funding and infrastructure development should be prioritized, with equitable distribution of research resources and funding intensity. It is vital to strengthen policy research and regulation to develop comprehensive biosecurity policies that address current and future threats, including guidelines for the safe handling of new and emerging pathogens. Regulations should be regularly reviewed and updated to reflect advances in technology and changes in global health security. Organizations should foster exchanges and cooperation among government agencies, academic institutions, nonprofit organizations, and other entities to complement each other’s strengths. Furthermore, a proactive approach to optimizing and integrating research directions across disciplines should be undertaken. Researchers should focus on promoting cutting-edge research areas, such as emerging technological innovations, security issues, changes in global health and security, interdisciplinary intersections, artificial intelligence, automation, and diversified research approaches.

## Author contributions

**Conceptualization:** Sunyun Qi, Hua Gu.

**Data curation:** Sunyun Qi, Siyuan Chen.

**Formal analysis:** Siyuan Chen.

**Investigation:** Geert Molenberghs.

**Project administration:** Geert Molenberghs.

**Resources:** Qifeng Zhang.

**Software:** Qifeng Zhang.

**Supervision:** Dries De Witte.

**Validation:** Hua Gu.

**Visualization:** Hua Gu.

**Writing – original draft:** Yanchao Gao.

**Writing – review & editing:** Yanchao Gao.
